# 529. Deceased Burn Patients as Organ Donors: Post-transplant Outcomes

**DOI:** 10.1093/jbcr/irag033.226

**Published:** 2026-04-06

**Authors:** Hunter Tessman, Lisa Heideman, Scott Sander, Duncan Nickerson, Julia C Slater

**Affiliations:** The University of Kansas Health System Burnett Burn Center, Shawnee, KS; The University of Kansas Health System Burnett Burn Center, Kansas City, KS; Midwest Transplant Network, Kansas City, KS; The University of Kansas Health System Burnett Burn Center, Kansas City, KS; The University of Kansas Health System Burnett Burn Center, Kansas City, KS

## Abstract

**Introduction:**

Currently, there are 105 459 patients on the transplant waiting list in the United States. By contrast, only 48 149 (~46%) patients received a transplant during 2024. Currently, there is high demand and low supply of organs, leaving many transplant recipients perpetually on the waiting list never able to receive an organ donation. Previous studies have been done on organ donation by deceased burn patients, but there are still a small number of studies on the topic. Burn patients with unsurvivable injuries are often overlooked as potential organ donors due to the multi-system organ failure and sepsis that results from many burn injuries, preventing their organs from being transplantable. We reviewed our center’s experience with referral of burn patients experiencing brain death or cardiac death for potential organ donation over a one-year period.

**Methods:**

A review of available records between 2023 and 2024 yielded 3 deceased burn patients who were evaluated for potential organ donation. There were no exclusion criteria. A descriptive review of the characteristics of these cases was undertaken.

**Results:**

Of the 3 burn patients declared brain dead or switched to Comfort Measure Only (CMO) care and referred for potential organ donation, 3 underwent organ retrieval with 2 having organs subsequently transplanted to recipients. The one patient who did not undergo donation was excluded due to dark purple kidneys observed upon removal. Our study population included a mean age 28.3 years old, mean Total Burn Surface Area (TBSA) of 55%, mean time to decision to withdraw care of 79.95 hours, 2 males, and 1 female patient. Two burn injuries were by a thermal mechanism, while 1 was electrical. Two patients were eligible donors after cardiac death (DCD), while 1 patient was an eligible donor after brain death (DBD). Two patients had at least 1 burn surgery, while 1 patient did not.

**Conclusions:**

DBD burn patient donors usually have better outcomes than DCD burn patient donors, as they can avoid the sepsis and multi-system organ dysfunction that typically accompanies DCD donors. The patient that was unable to undergo transplant was a DCD donor suffering metabolic acidosis, lactic acidosis, and acute hypoxic respiratory failure. Broadly, there remains a significant un-met donor organ need.

**Applicability of Research to Practice:**

When death is the expected outcome for a burn-injured patient, it is not always top-of-mind for caregivers or family members to consider potential organ donation. Nonetheless, all members of the burn care team should be reminded to consider the possibility of organ donation. Provision of training workshops regarding strategies for the tactful introduction of the topic could prove useful.

**Funding for the study:**

N/A.

